# Cytoprotective Effects of Hydrogen Sulfide in Novel Rat Models of Non-Erosive Esophagitis

**DOI:** 10.1371/journal.pone.0110688

**Published:** 2014-10-21

**Authors:** Oksana Zayachkivska, Olena Havryluk, Nazar Hrycevych, Nazar Bula, Oksana Grushka, John L. Wallace

**Affiliations:** 1 Physiology Department, Danylo Halytsky Lviv National Medical University, Lviv, Ukraine; 2 Department of Pathology, Danylo Halytsky Lviv National Medical University, Lviv, Ukraine; 3 Central Scientific Research Laboratory, Danylo Halytsky Lviv National Medical University, Lviv, Ukraine; 4 Department of Physiology & Pharmacology, University of Calgary, Calgary, Alberta, Canada; University of Louisville, United States of America

## Abstract

Non-erosive esophagitis is a chronic inflammatory condition of the esophagus and is a form of gastroesophageal reflux disease. There are limited treatment options for non-erosive esophagitis, and it often progresses to Barrett’s esophagus and esophageal carcinoma. Hydrogen sulfide has been demonstrated to be a critical mediator of gastric and intestinal mucosal protection and repair. However, roles for H_2_S in esophageal mucosal defence, inflammation and responses to injury have not been reported. We therefore examined the effects of endogenous and exogenous H_2_S in rat models of non-erosive esophagitis. Mild- and moderate-severity non-erosive esophagitis was induced in rats through supplementation of drinking water with fructose, plus or minus exposure to water-immersion stress. The effects of inhibitors of H_2_S synthesis or of an H_2_S donor on severity of esophagitis was then examined, along with changes in serum levels of a pro- and an anti-inflammatory cytokine (IL-17 and IL-10, respectively). Exposure to water-immersion stress after consumption of the fructose-supplemented water for 28 days resulted in submucosal esophageal edema and neutrophil infiltration and the development of lesions in the muscular lamina and basal cell hyperplasia. Inhibition of H_2_S synthesis resulted in significant exacerbation of inflammation and injury. Serum levels of IL-17 were significantly elevated, while serum IL-10 levels were reduced. Treatment with an H_2_S donor significantly reduced the severity of esophageal injury and inflammation and normalized the serum cytokine levels. The rat models used in this study provide novel tools for studying non-erosive esophagitis with a range of severity. H_2_S contributes significantly to mucosal defence in the esophagus, and H_2_S donors may have therapeutic value in treating esophageal inflammation and injury.

## Introduction

Gastroesophageal reflux disease (GERD) is a chronic, acid-related condition with extensive global, social and economic impacts [1]–[3]. Considerable progress has been made in understanding the pathogenesis of this disorder. This includes elucidation of the chain of events related to increased frequency of transient lower esophageal sphincter relaxations, abnormal esophageal and gastric peristalsis, decreased esophageal epithelial barrier function and visceral hypersensitivity [4]–[6]. The endoscopic-negative type of GERD, known as nonerosive reflux disease, is seen twice as frequently as the endoscopic-positive type. It can be associated with a diverse set of extra-esophageal conditions, including asthma, reflux laryngitis and periodontitis [7]–[9]. In addition, the conventional treatment for non-erosive reflux disease with gastric acid suppressing medications has been associated with an increased incidence of abnormal microbiota and malignancy [10]. According to a recent study, gastric acid is a strong activator a number of autoprotective mechanisms, including proliferation and differentiation, as well as the production of anti-inflammatory cytokines, growth factors and endogenous antioxidants [4], [11], [12]. Conditions such as Barrett’s esophagus, esophageal stricture and esophageal adenocarcinoma, the latter being identified as the most pernicious cancer of the gastrointestinal tract, have sharply risen in incidence over the last decade [9], [13].

The diagnostic and therapeutic approaches to non-erosive reflux disease are limited, in part because of the difficulties of investigating the pathogenesis of this condition in humans [2], [8]. Development of animal models of non-erosive reflux disease would assist in delineating the early events in its pathogenesis, which would hopefully lead to improved therapies. Indeed, several important advances have been made with respect to understanding the early biochemical and molecular mechanisms of ulceration and healing in other parts of the GI tract [12], [14]–[16].

Postprandial hyperglycemia is a risk factor for acid reflux and the development of non-erosive esophagitis. During the postprandial period, gastric reflux is increased [2], [17]. Several animal and human studies suggest that this is responsible for initiating esophageal mucosal injury and the development of dysmotility [13], [18], [19]. Moreover many metabolic disorders and diet-related chronic diseases appear to play key roles in the pathogenesis of GERD and non-erosive reflux disease [17], [20], [21]. Indeed, experimental long-term postprandial hyperglycemia contributes to impairment of the esophageal barrier function [3], . This impairment includes esophageal ischemia and hypoxia secondary to microvascular changes and peroxynitrite-mediated endothelial and enteric neuron damage [24], [25].

In recent years, H_2_S has been shown to exhibit a number of beneficial effects in the GI tract, including increasing mucosal resistance to damage induced by nonsteroidal anti-inflammatory drugs [14], [26]–[28] and ischemia-reperfusion [29], and acceleration of healing of mucosal ulcers [30], [31]. Endogenous H_2_S is produced from L-cysteine, with the enzymes cystathionine γ-lyase (CSE) and cystathionine β-synthase (CBS) representing two of the major pathways [31]–[33]. Suppression of endogenous H_2_S synthesis has been shown to impair gastric and colonic mucosal defence, and to impair healing of ulcers [26], [27], [34], [35]. The role of H_2_S in maintenance of esophageal mucosal integrity and healing has not been examined. Thus, the present study was designed to examine the effects of H_2_S on esophageal mucosal integrity and its possible contribution of esophagitis. The models of esophagitis used combined two elements known to contribute to esophageal injury: hyperglycemia [36] and exposure to restraint-stress [13], [37]. In addition to studying the role of H_2_S in esophageal injury, we examined the effects of L-tryptophan (L-Trp), since it has been reported to have protective effects in the esophagus [1], [24].

## Materials and Methods

### Animals

All experiments were carried out using rats weighing 180–220 g in accordance with the norms of the European Convention for the Protection of Vertebrate Animals Used for Experimental and Other Scientific Purposes (1986), as well as the Committee on Bioethics of Lviv National Medical University (protocol No 5, 17.05.2010). Male, wistar rats were maintained under a constant 12 h light/dark cycle and an ambient temperature of 21–23°C. All rats were fed by standard diet and were kept in raised mesh-bottom cages to prevent coprophagy. The rats were deprived of food for 18 h before the experiment, but had free access to water. The rats were anesthetized with an intramuscular injection of ketamine (60 mg/kg; Biovet, Ukraine). Six to seven rats were used in each group.

### Induction of Esophagitis

Moderate severity esophagitis was inducing hyperglycemia [23], [36]. This was accomplished by providing the rats *ad libitum* access to fructose-water (200 g/L) for 28 days, versus the control group with tap water access. To induce more severe esophagitis, the hyperglycemia model was combined with the esophageal lesion model of Takagi et al. [37] that involves short-term exposure to water-immersion stress. The rats were placed in restraint cages and immersed vertically to the level of the xiphoid process in a water bath of 23° for 3.5 h [37]. The initial and final body weights were recorded. Blood glucose concentrations were measured daily by glycometer (Achtung TD-4207, Germany) using a blood sample from the tail vein.

### Role of Hydrogen Sulfide

The second area of our study was aimed at determining the role of H_2_S in modulating the severity of esophagitis in the models used. Groups of 6–7 rats each were treated orally with vehicle, an inhibitor of CSE (L-propargylglycine; PAG; 25 mg/kg), an inhibitor of CBS (O-carboxymethylhydroxylamine; CHH, 20 mg/kg), or with an H_2_S donor (NaHS; 100 µmol/kg). Esophageal integrity was scored 4 hours after the last treatment dose using criteria described below.

Additionally, groups of 6–7 rats each that had received the fructose-supplemented water with or without exposure water-immersion stress were similarly pre-treated (at −30 min) with the H_2_S-synthesis inhibitors or the H_2_S donor. These doses of PAG and CHH have been shown to significantly inhibit CSE and CBS activities in rats [34], [35]. As a positive control, some rats were treated orally with L-Trp at a dose of 50 mg/kg, since this has previously been shown to exert protective effects in experimental esophagitis [1], [24]. Esophageal integrity was scored 4 hours after the initial drug/vehicle administration, using criteria described below.

Immediately after the termination of experiment, venous blood samples were drawn from the abdominal vein and placed into EDTA-containing vials and used for the determination of levels of interleukin-10 (IL-10) and interleukin-17 (IL-17). The blood samples were centrifuged at 3500 rpm for 10 min at a temperature of 15°C. The plasma was collected with a micropipette and stored in −60°C until the ELISA assay was performed according to the manufacturer’s instructions (Multi-Analyte ELISArray Kit; Vector-Best, Russia). The intensity of the colour reaction was estimated using a GBG Stat-Fax 303 Plus Microstrip Reader (Shaker Stat-Fax 2200 Awareness Technology, Inc., Palm City, FL, USA) at 450 nm and 620–655 nm, respectively.

### Evaluation of Damage

The rat esophagus was removed immediately after sacrifice and cut with scissors in the longitudinal direction from the gastresophageal junction to the pharynx. The mucosal surface was gently washed with phosphate-buffered (pH 7.4) saline. Samples of the mucosa from the lower third of the esophagus were excised at the region 2 mm below the lower esophageal sphincter that separates the forestomach from the oeosphagus. They were fixed in 10% formalin and embedded in parrafin. Serial sections of 5-µm thickness were stained with hematoxylin/eosin. The sections were blindly evaluated by two individuals and their scores were averaged. The scoring included three components: loss of epithelium (0 - none, 1– minimal pre-ulcerative changes and splitting, 2 - erosion, 3– ulceration), vascular changes (0 - none; 1 - edema, 2 - submucosal vascular dilation, 3– perivascular haemorrhage) and intraepithelial leukocyte infiltration (0 - none, 1 - mild, 2 - moderate, 3– severe).

### Materials

L-tryptophan, L-PAG, CHH and NaHS were from Sigma-Aldrich (St. Louis, MO, USA). The Multi-Analyte ELISArray Kits for measurement of IL-10 and IL-17 were obtained from Vector-Best (Novosibirsk, Russia).

### Statistical Analysis

Results are presented as the mean **±** standard error of the mean (SEM). For comparisons of two groups of data, the Student’s t-test was used. In all other circumstances, the data were analyzed by a one-way analysis of variance followed by the Newman-Keuls test. An associated probability of less than 5% was considered significant.

## Results

### Inhibition of H_2_S Synthesis Caused Mild Esophageal Inflammation in Healthy Rats

**Figure 1:**
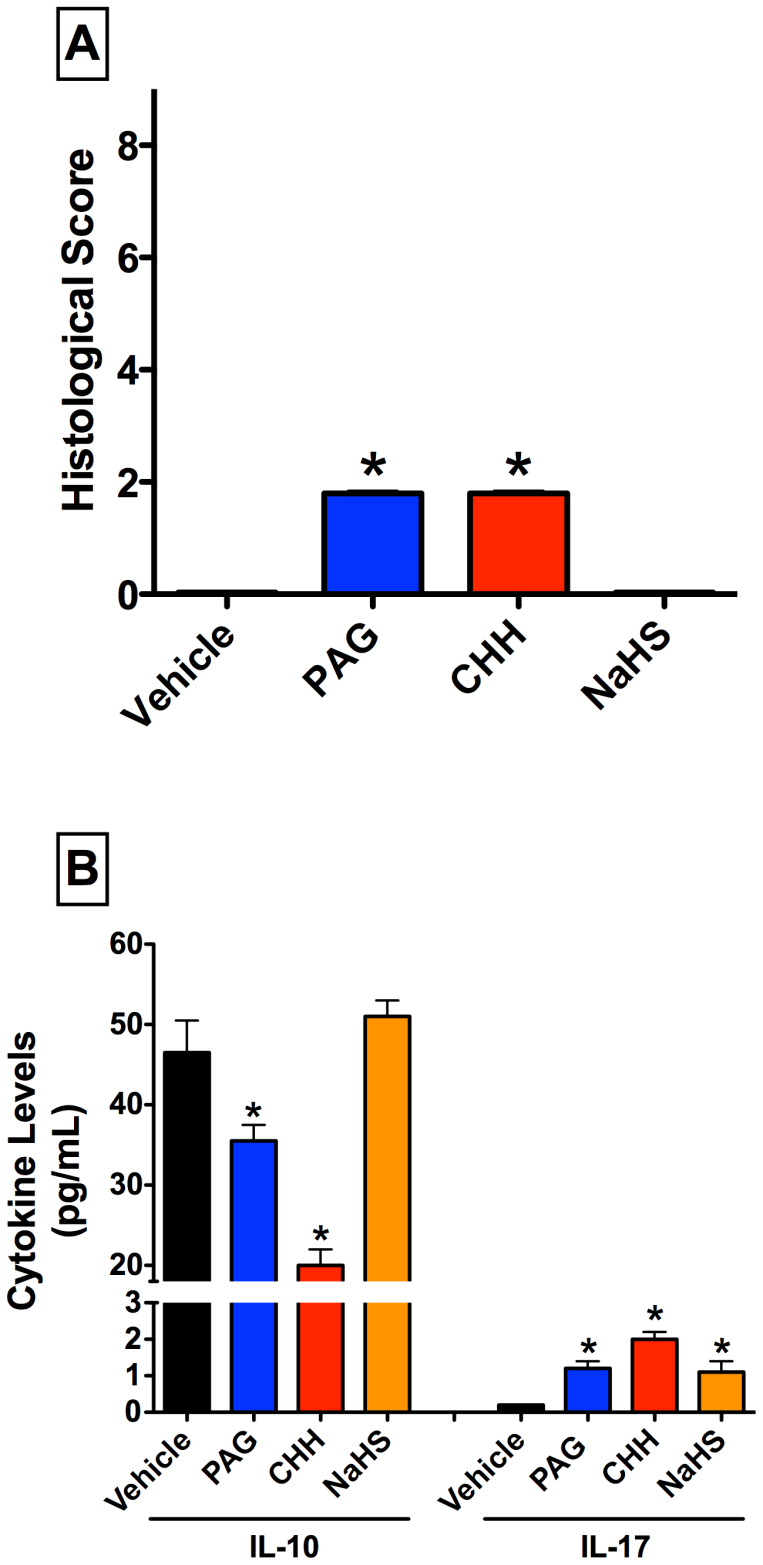
Treatment of healthy rats with inhibitors of hydrogen sulfide (H_2_S) synthesis produces negligible esophageal damage, but significantly alters serum levels of IL-10 and IL-17. Panel A: Small, but statistically significant changes in the histological score of lower esophageal integrity were observed following administration of either of the inhibitors of H_2_S synthesis (PAG, L-propargylglycine and CHH, O-carboxymethylhydroxylamine). The changes induced by the H_2_S inhibitors were limited to mild mucosa inflammation. Administration of the inhibitors of H_2_S synthesis also resulted in significant decreases in serum IL-10 levels, while the H_2_S donor, NaHS, had no effect. Small but significant increases in IL-17 levels were observed following administration of PAG, CHH and NaHS. Bars represent the mean ± SEM of at least 6 rats/group. *p<0.05 versus the vehicle-treated group (one-way ANOVA and Neuman-Keuls test).

Healthy rats treated with vehicle exhibited normal esophageal appearance, with histological scores of zero. Treatment with inhibitors of H_2_S synthesis (PAG or CHH) resulted in modest effects on the esophagus, with irregular subepithelial exudates, submucosal vascular dilation and mild leukocyte infiltration in the esophageal mucosa. There was no detectable mucosal damage. The histological scores ranged between 1 and 2 (on a 0 to 9 scale) (Fig. 1). These findings are similar to what is observed in human microscopic reflux esophagitis, and would be assigned a Grade M in accordance with the updated Los Angeles classification [2].

Despite evidence for only minor esophageal inflammation following suppression of endogenous H_2_S synthesis, there were marked changes in serum levels of anti-inflammatory and a pro-inflammatory cytokines. Treatment with PAG and CHH both resulted in significant decreases in serum IL-10 and increases in IL-17 (Fig. 1). In contrast, treatment with NaHS, which spontaneously releases H_2_S, did not affect serum IL-10 levels, while IL-17 levels were significantly increased over those observed in vehicle-treated rats (note: in vehicle-treated rats, the serum IL-17 levels were non-detectable or very close to the limit of detectability).

### H_2_S Modulated Esophageal Injury and Inflammation Induced by Hyperglycyemia

**Figure 2:**
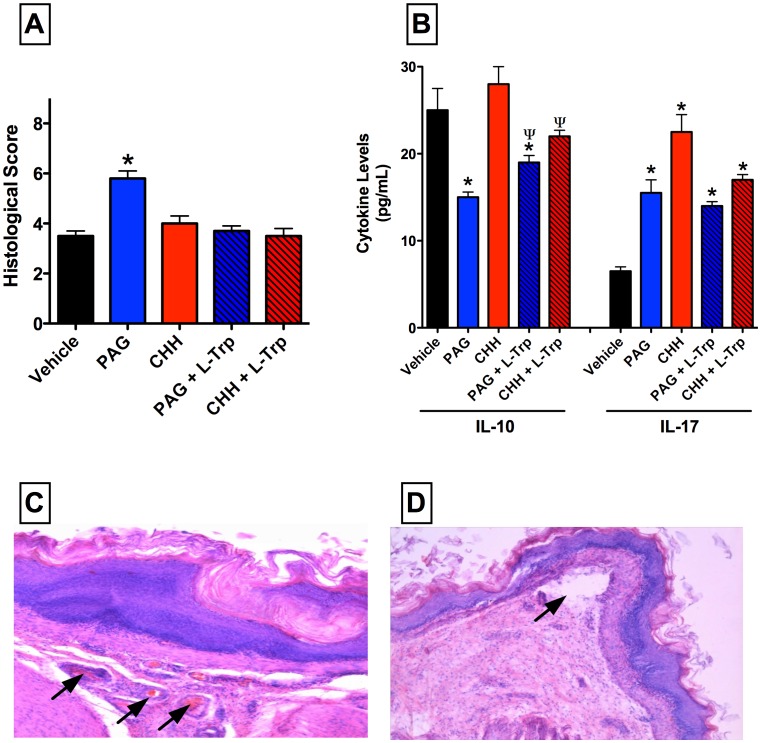
Administration of an inhibitor of hydrogen sulfide (H_2_S) synthesis via cystathionine γ-lyase (L-propargylglycine; PAG) exacerbates esophageal injury/inflammation in hyperglycemic rats. Administration of PAG (25 mg/kg) resulted in a significant increase in the histological score of esophageal injury (panel A). PAG administration also reduced serum IL-10 and increased serum IL-17 levels. The effects of PAG on esophageal damage and serum IL-10 were reversed by co-adminstration of L-tryptophan (L-Trp). An inhibitor of another pathway of H_2_S synthesis (CHH; O-carboxymethylhydroxylamine; 20 mg/kg) had no effect on esophageal injury, but produced similar changes to serum IL-10 and IL-17 levels as were seen in PAG-treated rats. Panel C shows the irregular hyperemia, stasis (arrows) and perivascular diapedesis that was observed in rats that on the fructose-supplemented drinking water that were treated with PAG. Panel D illustrates that this treatment also resulted in localized detachments of the epithelium from the basement membrane and destructive changes to the epithelial plate (arrow). X200 (hematoxylin and eosin staining). Bars represent the mean ± SEM of at least 6 rats/group. *p<0.05 versus the vehicle-treated group; ^ψ^p<0.05 versus the corresponding group not treated with L-tryptophan (one-way ANOVA and Neuman-Keuls test).

Rats consuming drinking water supplemented with fructose for 28 days exhibited a significant elevation of blood glucose levels from a mean of 5.8±0.2 mmol/L to 6.4±0.2 mmol/L (p<0.05), and an 8–10% gain in body weight over that of the control rats. Examination of the esophagus of the hyperglycemic rats revealed significant inflammatory changes, and the histological score was significantly elevated above that of healthy rats (Fig. 2). The histological changes induced by hyperglycemia included elongation of papillae of esophageal mucosa with dilated vascular channels at the tips of the papillae and epithelial desquamation to the lumen, increased mitoses in epitheliocytes, spongiosis and swelling of keratinocytes in the superficial layer, proliferation of lamina propria of epithelium and mild epithelial leucokyte infiltration (Fig. 2).

Administration of L-PAG, but not CHH, resulted in a significant worsening of esophagitis in the hyperglycemic rats. The damage was characterized by signs of irregular hyperaemia, vascular stasis, perivascular diapedesis with microthrombi in subepithelial vessels and perivascular hemorrhage, as well as splitting of epithelium and its desquamation (Fig. 2). There was also subepithelial edema combined with moderate leukocyte intraepithelial infiltration. The exacerbation of esophagitis by PAG was completely reversed by co-administration L-Trp.

There was a similar pattern of changes in serum cytokine levels as observed in the healthy rats. Thus, treatment with PAG reduced serum levels of IL-10 and increased serum levels of IL-17. Treatment with L-Trp, which attenuated the detrimental effects of PAG in the esophagitis model, modestly increased serum IL-10 levels but did not affect serum IL-17 levels (Fig. 2).

### H_2_S Protects against Severe Esophagitis Induced by Hyperglycyemia Plus Stress

**Figure 3:**
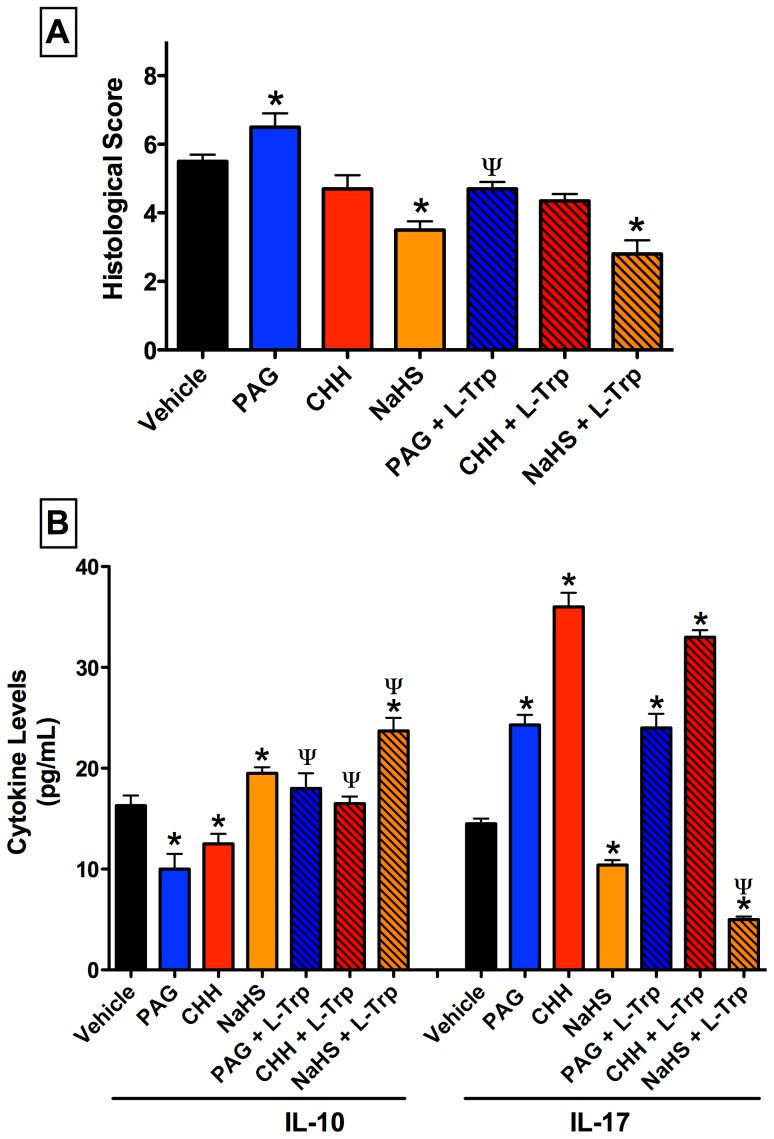
Administration of an inhibitor of hydrogen sulfide (H_2_S) synthesis via cystathionine γ-lyase (L-propargylglycine; PAG) increases esophageal injury/inflammation in hyperglycemic rats subjected to water-immersion stress. The exacerbation of histological damage by PAG (25 mg/kg) was reversed by co-administration of L-tryptophan (L-Trp). Both inhibitors of H_2_S synthesis significantly reduced serum IL-10 and increased serum IL-17, and co-administration of L-Trp diminished these effects. The H_2_S donor, NaHS, significantly increased IL-10 and reduced IL-17, and the combination of NaHS and L-Trp produced significantly greater changes in levels of these two cytokines. Bars represent the mean ± SEM of at least 6 rats/group. *p<0.05 versus the vehicle-treated group; ^ψ^p<0.05 versus the corresponding group not treated with L-Trp (one-way ANOVA and Neuman-Keuls test).

**Figure 4:**
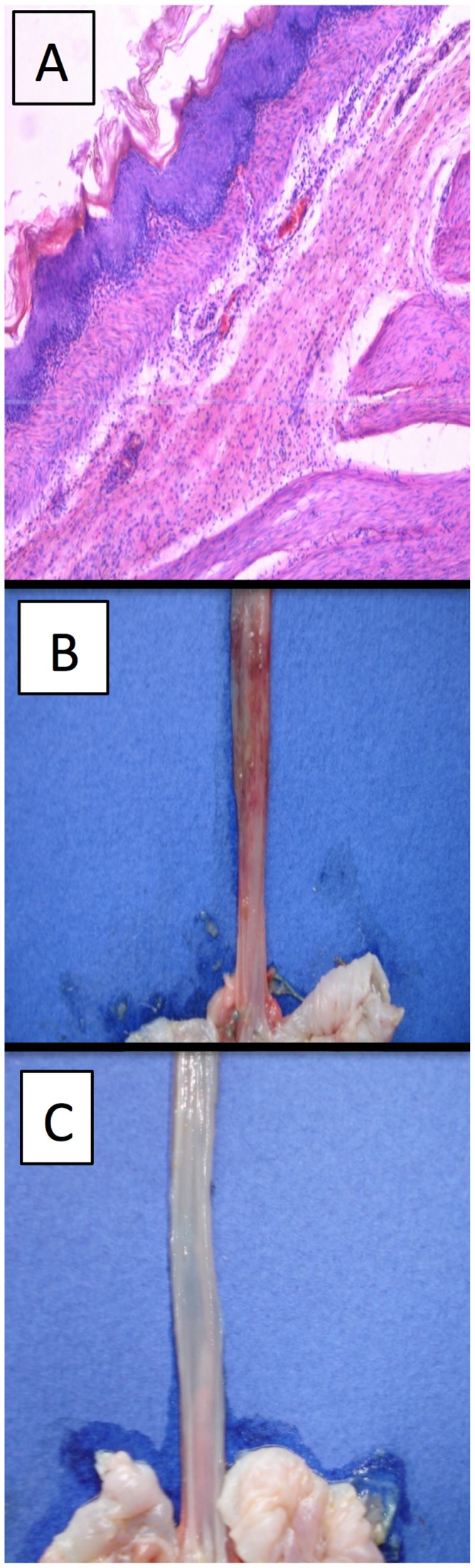
Esophageal lesions with signs of nonerosive microscopic esophagitis induced by water-immersion stress after 28 days of consumption of fructose-supplemented drinking water. Panel A: Pretreatment with an inhibitor of cystathionine γ-lyase (L-propargylglycine; PAG) resulted in extensive esophageal inflammation, with profound submucosal leukocyte infiltration. Panel B: Macroscopic appearance of the lower esophagus of rat treated with PAG, with extensive perivascular hemorrhage. Panel C: Macroscopic appearance of lower esophagus of rat treated with PAG and L-tryptophan, the latter providing a protection against the detrimental effects of PAG.

Severe mucosal lesions developed in the esophagus of vehicle pre-treated, hyperglycemic rats subjected to water-immersion stress (Fig. 3). Pre-treatment with PAG, but not CHH, significantly increased the severity of the esophagitis (Fig. 3 and 4). This included esophageal subepithelial vascular changes such as oedema, submucosal vascular dilation, perivascular haemorrhage, and perivascular diapedesis combined with elevated intraepithelial leukocyte infiltration (Fig. 4). Administration of the H_2_S donor, NaHS, significantly reduced the severity of esophagitis. Co-administration of L-Trp with PAG significantly reduced the severity of esophageal damage observed when only the latter was administered. The combination of NaHS and L-Trp reduced histological changes in the esophageal mucosa of rats dramatically, with sharply decreased inflammation, subepithelial edema, and less epithelial disorganization. Macroscopically, there was a clear protective effect observed when L-Trp was co-administered with PAG, with complete prevention of the hemorrhagic changes that was observed when only PAG was administered (Fig. 4B and 4C).

Changes in serum cytokine levels were similar to those observed in the other models of esophagitis (Fig. 3). Treatment with PAG or CHH each resulted in decreased serum levels of IL-10 and increased serum levels of IL-17, while NaHS had the opposite effects. Co-administration of L-Trp with PAG or CHH resulted in restoration of serum IL-10 levels to those of vehicle-treated rats, but had no effect on serum IL-17 levels. When L-Trp was co-administered with NaHS, serum IL-10 levels were significantly increased and serum IL-17 levels were significantly decreased.

### Changes in Serum Cytokines Correspond to Severity of Esophagitis

**Figure 5:**
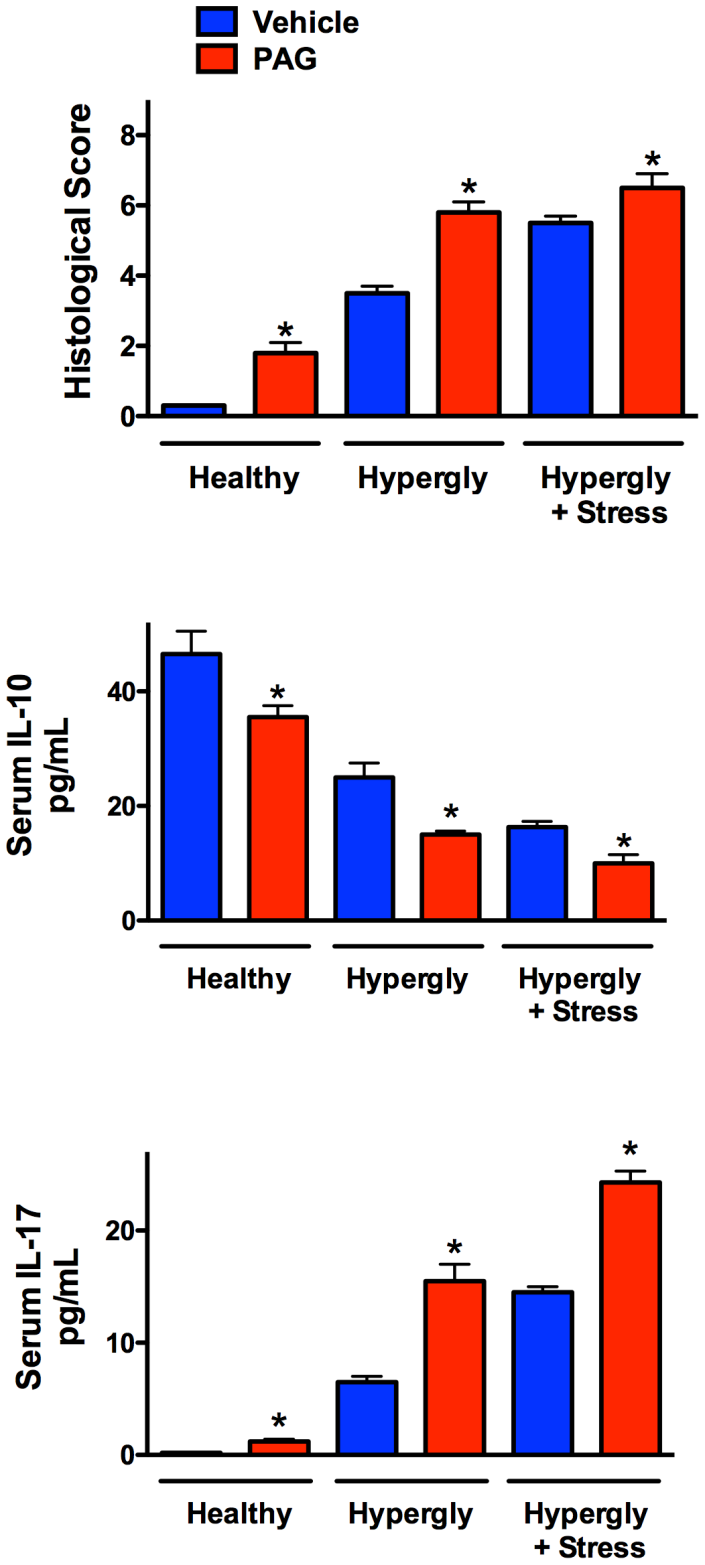
Summary of some of the key observations in models of mild, moderate and severe esophagitis in rats, and the effects of an inhibitors of hydrogen sulfide synthesis via cystathionine γ-lyase (L-propargylglycine; PAG). In healthy rats and in the rats with hyperglycemia- or hyperglycemia+stress-induced esophagitis, acute administration of PAG caused a significant exacerbation of esophageal inflammation. Serum levels of IL-10 decreased with the severity of esophagitis, and this was further enhanced in rats treated with PAG. In contrast, serum levels of IL-17 increased sharply in animal with esophagitis, in parallel with the severity of the disease, and administration of PAG caused further increases in all three models. *p<0.05 versus the corresponding vehicle-treated group (Student’s t-test).

Figure 5 illustrates the pattern of changes in histological scores and serum cytokine levels in the three models used in this study. The histological scores for esophagitis increased from healthy rats to the hyperglycemia model, and were further increased in the hyperglycemia+stress model. In each model, there was a significant increase in the severity of esophagitis when PAG (but not CHH) was administered.

The increases in histological scores in the three models were mirrored by increases in serum IL-17, and decreases in serum IL-10 levels. PAG treatment was consistently associated with decreased serum IL-10 levels and increased IL-17 levels.

## Discussion

GERD is among the most common diseases in the world, and largely because of this high incidence, is one of the most expensive to treat. The non-erosive form of GERD has been increasing in frequency in recent years, mainly in parallel with the rise in incidence of various metabolic disorders (e.g., type-2 diabetes, metabolic syndrome, obesity) [3]. Non-erosive reflux disease accounts for over 75% of all global cases of GERD [3]. Recent studies have revealed a significant failure of proton pump inhibitor therapy in patients with metabolic disease-associated GERD [9], . These concerns highlight the importance of better understanding the pathogenesis of GERD and identifying effective means of preventing and treating this disorder [5], [21]. In the present study, suppression of synthesis of H_2_S, a gaseous mediator shown to play important roles in mucosal defence and healing in the stomach, small intestine and colon [14], [26]–[30], [34], was shown to exacerbate experimental non-erosive esophagitis. Administration of an H_2_S donor exerted significant protective effects in the models of non-erosive esophagitis. Inhibition of H_2_S synthesis was also associated with marked increases in serum levels of IL-10 (an anti-inflammatory cytokine) and decreased serum levels of IL-17 (a pro-inflammatory cytokine), while the H_2_S donor had the opposite effect.

The role of H_2_S as a mediator of inflammation and mucosal integrity in the esophagus has not previously been reported. However, there is substantial evidence that H_2_S is produced throughout the GI tract and it participates in many physiological functions in addition to promoting mucosal integrity, reducing mucosal inflammation and accelerating healing of ulcers [28]–[30], [34], [38]. Accleration of ulcer healing is most likely related to the ability of H_2_S to promote angiogenesis [30], [39], while prevention of tissue injury may be related to the ability of H_2_S to maintain mucosal blood flow [29], [38], stimulate bicarbonate secretion [40], [41], stimulate mucus secretion [42], inhibit leukocyte adherence to the vascular endothelium [43], scavenge free radicals [44], promote resolution of inflammation by increasing neutrophil apoptosis [45] and differentiation of macrophages to the M2 phenotype [46], and suppress production of pro-inflammatory cytokines (including IL-1, IL-2, IL-8 and TNFα) [47]–[50], while maintaining or increasing production of IL-10 [47]–[50]. Suppression of IL-8 production in keratinocytes by H_2_S has been reported to be a consequence of diminished IL-17 production, as IL-17 can induce IL-8 production [51], [52]. Thus, diseases characterized by an important role for IL-17 may be rationale targets for H_2_S-based therapies.

With respect to the effects of inhibition of H_2_S synthesis, it was notable that administration of PAG consistently resulted in an exacerbation of esophageal inflammation/injury, while CHH had no such effect on the histological scores. PAG is an inhibitor with selectivity for the enzyme cystathione γ-lyase (CSE), while CHH inhibits cystathione β-synthase (CBS) and cysteine aminotransferase [53]. Thus, our results suggest a predominant role of CSE in producing H_2_S in the esophageal tissue, which is consistent with this enzyme playing a more important role than CBS in producing H_2_S in other parts of the GI tract [29], [30], [34], [38]. However, similar alterations in serum levels IL-10 and IL-17 were observed with CHH and PAG. This suggests that the alteration of cytokine levels was likely a systemic effect, not necessarily a reflection of or contributing to the observed changes in esophageal inflammation and injury. Nevertheless, the changes in basal cytokine levels did correlate well with the degree of histologically confirmed inflammation in the espophagus.

There is growing evidence that over-consumption of sugars contributes to esophageal motor dysfunction [54], [55], and in turn to the increase in acid reflux and esophagitis in individuals with metabolic disorders. Hyperglycemia triggers relaxation of the lower esophageal sphincter [55], thereby promoting acid reflux. The most commonly used therapy for gastric acid reflux, proton pump inhibitors, have an increased failure rate in people with type II diabetes [54]. There is also evidence that the increase in reflux esophagitis in type II diabetes occurs via elevated oxidative stress [54], [55]. In the present study, we demonstrated that this clinical scenario could be mimicked in the laboratory by inducing hyperglycemia in rats through supplementation of drinking water with fructose. It has been proposed that post-prandial hyperglycemia initiates a shift in redox systems, characterized by cellular membrane injury by free radicals, as well as reduced circulating homocysteine levels [56]. These changes are attributed to proliferation and transformation of the normal squamous epithelium of the esophagus into gastric-type mucosa, a pre-malignant condition [12]. Relevant to the present study, homocysteine is one of the precursors for H_2_S synthesis, which we demonstrate to play a critical role in maintenance of esophageal mucosal integrity. Recent studies have suggested that acute inflammation is a primary driver of impaired esophageal mucosal integrity, with subsequent loss of “barrier function”, resulting in dramatically increased iNOS and COX-2 expression [17], [24], [57]–[59]. We previously demonstrated increased production of nitric oxide metabolites and iNOS activity, as well as altered eicosanoid synthesis in experimental non-erosive esophagitis induced by experimental diabetes, hyperglycemia or restraint-stress in rats [17], [23], [24], [57], [58]. H_2_S has been implicated as a regulator of both nitric oxide and eicosanoid ynthesis [28], [34], [60].

We conclude that H_2_S biosynthesis contributes significantly to regulation of esophageal mucosal defence and inflammation, and reduced production of this mediator leads to exacerbation of esophagitis. The marked reduction of esophageal damage and inflammation by an H_2_S donor, alone or in combination with L-Trp, suggests that agents such as these could be exploited in development of novel, effective therapies for non-erosive esophagitis. Indeed, several recent studies provide clear evidence for the effectiveness and safety of H_2_S-based therapeutics for a range of disorders, including those characterized by injury and inflammation of the digestive tract [61].
